# Lung Ultrasound Characteristics in Neonates With Positive Real Time Polymerase Chain Reaction for SARS-CoV-2 on a Tertiary Level Referral Hospital in Mexico City

**DOI:** 10.3389/fped.2022.859092

**Published:** 2022-04-08

**Authors:** Daniel Ibarra-Ríos, Andrea Constanza Enríquez-Estrada, Eunice Valeria Serpa-Maldonado, Ana Luisa Miranda-Vega, Dina Villanueva-García, Edna Patricia Vázquez-Solano, Horacio Márquez-González

**Affiliations:** ^1^Neonatology Department, National Institutes of Health, Hospital Infantil de México Federico Gómez, Mexico City, Mexico; ^2^Clinical Investigation Department, National Institutes of Health, Hospital Infantil de México Federico Gómez, Mexico City, Mexico

**Keywords:** SARS-CoV-2, lung ultrasound, POCUS, newborn, COVID-19

## Abstract

**Introduction:**

Acute respiratory syndrome secondary to SARS-CoV-2 virus infection has been declared a pandemic since December 2019. On neonates, severe presentations are infrequent but possible. Lung ultrasound (LUS) has been shown to be useful in diagnosing lung involvement and following up patients, giving more information, and reducing exposure compared to traditional examination.

**Methods:**

LUS was performed after the diagnosis of SARS-CoV-2 infection with respiratory Real Time Polymerase Chain Reaction RT-PCR with portable equipment protected with a silicone sleeve. If hemodynamic or cardiology consultation was necessary, a prepared complete ultrasound machine was used. Ten regions were explored (anterior superior and inferior, lateral, and posterior superior and inferior, right and left), and a semiquantitative score (LUSS) was calculated. Disease severity was determined with a pediatric modified score.

**Results:**

Thirty-eight patients with positive RT-PCR were admitted, 32 (81%) of which underwent LUS. Included patients had heterogenous diagnosis and gestational ages as expected on a referral neonatal intensive care unit (NICU) (median, ICR: 36, 30–38). LUS abnormalities found were B-line interstitial pattern 90%, irregular/interrupted/thick pleural line 88%, compact B-lines 65%, small consolidations (≤5 mm) 34%, and extensive consolidations (≥5 mm) 37%. Consolidations showed posterior predominance (70%). LUSS showed a median difference between levels of disease severity and ventilatory support (Kruskal–Wallis, *p* = 0.001) and decreased with patient improvement (Wilcoxon signed-rank test *p* = 0.005). There was a positive correlation between LUSS and FiO_2_ needed (Spearman *r* = 0.72, *p* = 0.01). The most common recommendation to the attending team was pronation (41%) and increase in positive end expiratory pressure (34%). Five patients with comorbidities died. A significant rank difference of LUSS and FiO_2_ needed between survivors and non-survivors was found (Mann–Whitney *U*-test, *p* = 0.005).

**Conclusion:**

LUS patterns found were like the ones described in other series (neonatal and pediatrics). Eighty-eight percent of the studies were performed with handheld affordable equipment. While there is no specific pattern, it varies according to gestational age and baseline diagnosis LUS, which were shown to be useful in assessing lung involvement that correlated with the degree of disease severity and respiratory support.

## Introduction

Acute respiratory syndrome secondary to SARS-CoV-2 virus infection has been declared a pandemic since December 2019. In Mexico, by the end of 2021, 3,988,916 cases were reported with 299,525 deaths. Regarding the pediatric population, infants <1 year old showed a case fatality rate of 2.1% (1). On neonates, severe presentations are infrequent but possible. Few reports demonstrate vertical transmission, and most cases are environmental. The most common symptoms are respiratory, and lung imaging is frequently abnormal (2).

At the beginning of the pandemic, computed tomography (CT) was suggested as a reliable tool in the diagnosis of interstitial pneumonia caused by COVID-19. Nevertheless, the cost, time consumption, need for intrahospital transfer, patient radiation, and proper decontamination of the equipment represented challenges. Several studies in adults have shown that the level of diagnostic agreement between lung ultrasound (LUS) and CT in the diagnosis of COVID-19 pneumonia is high (3). Giorno et al., in Brazil, described 34 confirmed pediatric patients including three newborns (one with CT). Both the findings and topography of lung compromise on the CT were consistent with the information obtained by LUS (4).

Lung ultrasound has been shown to be useful in diagnosing lung involvement and following up patients, giving more information, and reducing exposure compared to traditional examination. A LUS semiquantitative score (LUSS) allows functional applications, classifying the image into a score that implies less aeration with increasing scale (5).

Case series have been published in adults and pediatrics; in the case of neonates, few cases have been described.

## Materials and Methods

The study was done at a Pediatric Tertiary Level Referral Hospital restructured to receive COVID patients in Mexico City between April 2020 and December 2021 (6). Newborns with a positive real time polymerase chain reaction (RT-PCR) for the SARS-CoV-2 virus by nasal swab irrespective of the working diagnosis were included. Excluded patients spent a brief time in the neonatal intensive care unit (NICU) usually <48 h or were assessed in the emergency room and discharged without oxygen.

Lung ultrasound was performed after the diagnosis of SARS-CoV-2 infection with portable equipment protected with a silicone sleeve (Konted™, Beijing, China, linear 10.0 MHz) by an operator with full personal protective equipment. Image acquisition was obtained in a protected tablet by the same operator or at distance (Wi-Fi connection) by another expert operator as described by De Rose et al. (7). If hemodynamic or cardiology consultation was necessary, our NICU point-of-care ultrasound machine (Vivid™ *E90*, GE Medical Systems, Milwaukee, WI, United States, with a hockey stick probe 8–18 MHz) was prepared according to published guidelines (8).

Lung ultrasound semiology was defined as follows (5):

1. Pleural sliding: Horizontal to-and-fro motion of the pleural line.
2. A-line: Horizontal artifact equidistant to the pleural line, appreciated during the insonation of an aerated lung.
3. B-line: Laser-like vertical hyperechoic reverberation artifacts that arise from the pleural line, move synchronously with pleural sliding, and extend to the bottom of the screen without fading.
4. Interstitial syndrome: Defined as the presence of three or more well-spaced B-lines.
5. White lung: Coalescent B-lines that make A-lines disappear.
6. Consolidation: Subpleural hypoechoic region with shredding of the pleural line, with tissue-like echotexture and disappearance of A-lines. The threshold to distinguish small consolidations from extensive consolidations was arbitrarily defined as 5 mm.

**FIGURE 1:**
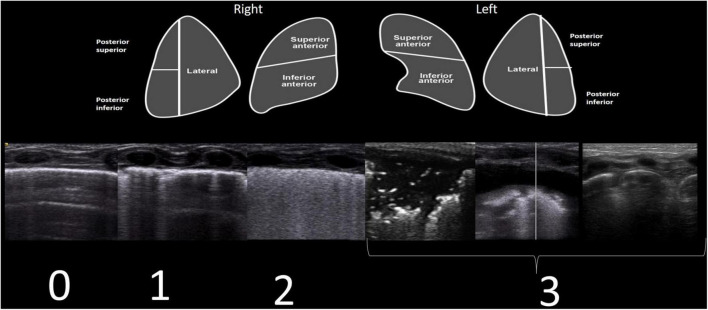
Each lung was divided into five areas (upper part of the figure). For each area, a score of 0–3 was established. LUSS corresponds to four different patterns (lower part of the figure). Scores were given as follows: 0 (defined by the presence of A-lines and pleural sliding); 1 (defined as the presence of three or more well-spaced B-lines); 2 (defined as the presence of coalescent B-lines or white lung); and 3 (defined as the presence of extended consolidations and pleural effusion or absence of lung in the case of lung malformations).

Ten regions were explored (anterior superior and inferior, lateral, and posterior superior and inferior, right and left). A semi-quantitative LUSS was calculated through both transverse and longitudinal scans, with the following scores: 0 (defined by the presence of A-lines and pleural sliding); 1 (defined as the presence of three or more well-spaced B-lines); 2 (defined as the presence of coalescent B-lines giving a white lung appearance); and 3 (defined as the presence of extended consolidations and pleural effusion or absence of the lung in the case of lung malformations) (Figure 1).

Additionally, on intubated patients, a transverse image on the anterior aspect of the neck at the level of the cricothyroid membrane was taken, demonstrating the tube between the thyroid lobes (air–mucosa interface with posterior reverberation and shadowing artifact), determining *in situ* placement. No diaphragmatic ultrasound was used.

Perinatal transmission was classified according to the WHO definitions available at https://www.who.int/publications/i/item/WHO-2019-nCoV-mother-to-child-transmission-2021.1.

The severity of the disease was classified with an adapted scale from the one reported by Parri et al. (9):

**Asymptomatic**, where all the following must be present:

1. No signs or symptoms.
2. AND negative chest X-ray.
3. AND absence of criteria for other cases.

In healthcare-associated infection, no change in respiratory status and support from baseline if the patient previously had indirect oxygen.

**Mild**, where any of the following must be present (AND absence of criteria for more severe cases):

1. Symptoms of upper respiratory tract infection.
2. AND absence of pneumonia at chest X-ray.

In healthcare-associated infection, respiratory support included new-onset indirect oxygen.

**Moderate**, all the following must be present (AND absence of criteria for more severe cases):

1. Sick appearing OR pneumonia at chest X-ray.
2. Respiratory support included CPAP with FiO_2_ ≤30%.

In healthcare-associated infection, respiratory support included patients intubated for a non-respiratory indication (surgical or congenital heart disease) with FiO_2_ ≤25%.

**Severe**, any of the following (AND absence of criteria as for critical case):

1. Oxygen saturation <90%.
2. OR difficulty in breathing or other signs of severe respiratory distress.
3. OR need for invasive respiratory support.

Respiratory support included mechanical ventilation FiO_2_ ≤60%.

In hospital-acquired infection, an increase in ≥10% FiO_2_ or pressures was considered if the patient was previously intubated.

**Critical**, any of the following must be present:

1. Respiratory support HFOV or mechanical ventilation with FiO_2_ ≥60%.
2. OR multi-organ failure.
3. OR new-onset shock, encephalopathy, myocardial injury or heart failure, coagulation dysfunction, acute kidney injury.

Follow-up studies were performed with our NICU point-of-care ultrasound machine (Vivid™ *E90*, GE Medical Systems, Milwaukee, WI, United States, with a hockey stick probe 8–18 MHz) once a negative test was obtained and the newborn entered the general ward (if the baby was not discharged early according to our NICU policies during the pandemic).

Statistical analysis was performed using SPSS Version 23.0 (IBM Corp.). Continuous variables were expressed as median ± interquartile ranges. Median difference of LUSS between levels of disease severity and pulmonary support were compared through the Kruskal–Wallis test. The correlation between LUSS and FiO_2_ was tested with the Spearman test. Median differences of baseline and follow-up LUSS were compared through the Wilcoxon signed-rank test.

## Results

Thirty-eight patients with positive RT-PCR were admitted, 32 (81%) of which underwent LUS. Fifty-three percent were female with a gestational age of 36 weeks, 30–38. Infection occurred at a corrected age of 38 weeks, 33–41. Patient baseline diagnosis was preterm infants 41% (13 patients, four with established bronchopulmonary dysplasia), term infant with respiratory distress 28% (nine patients), surgical patients 25% (eight patients), and congenital heart disease 6% (two patients). Eighty-one percent of patients were classified as respiratory upon arrival. Attributable new-onset respiratory symptoms included desaturation 75%, tachypnea 69%, respiratory distress 53%, apnea 34%, and rhinorrhea 16%. Cardiovascular symptoms included tachycardia 47% and hypotension 19%. Only four patients (12%) presented with fever (Table 1).

**TABLE 1:** Demographics, clinical, and laboratory findings during the acute phase of the SARS-CoV-2 infection.

Birth history	
Maternal age, years, median (IQR)	25 (20–30)
GA at birth, weeks, median (IQR)	36 (30–38)
Birth weight, g, median (IQR)	2,394 (1,117–2,861)
Cesarean section	25 (78)
Female (%)	17 (53)
Advanced resuscitation (%)	12 (37)
Chest compressions (%)	4 (12)
Intubated at birth (%)	8 (25)
5 min Apgar score (%)	8 (8–9)
Hypoxic Ischemic Encephalopathy (%)	5 (16)
Surfactant (%)	8 (25)
**Clinical characteristics during the acute phase of the** **SARS-CoV-2 infection**	
Corrected GA at infection, weeks, median (IQR)	38 (33–41)
Weight at infection, g, median (IQR)	2,615 (1,852–3,087)
**Clinical findings**	
Desaturation (%)	24 (75)
Tachypnea (%)	22 (69)
Respiratory distress (%)	17 (53)
Tachycardia (%)	15 (47)
Apnea (%)	11 (34)
Hypotension (%)	6 (19)
Rhinorrhea (%)	5 (16)
Fever (%)	4 (12)
**Laboratory findings**	
Hemoglobin, median (IQR)	14.6 (11.7–15.3)
Leukocytes, (/μL), median (IQR)	11,900 (9,750–15,200)
Lymphocytes, (/μL), median (IQR)	4,032 (3,300–5,502)
Neutrophils, (/μL), median (IQR)	5,936 (4,189–8,977)
Platelets, (/μL), median (IQR)	269,000 (141,000–336,000)
Creatinine, (μmol/L), median (IQR)	84 (35–117)
Aspartate Aminotransferase, (U/L), median (IQR),	25 (16.5–36)
Alanine Aminotransferase, (U/L), median (IQR)	56 (33–66)
Prothrombin time, s, median (IQR)	15.4 (12.15–18.25)
Activated partial thromboplastin time, s, median (IQR)	31.15 (27.7–42)
INR, median (IQR)	1.35 (1.05–1.6)
D-Dimer >1,500 mg/dl (%)	4 (12)
Antibiotics (%)	9 (28)
Hemodynamic support (%)	8 (25)
**Ventilatory support**	
Without oxygen (%)	4 (13)
Indirect oxygen (%)	8 (25)
Continuous airway positive pressure (%)	2 (6)
Mechanical ventilation (%)	15 (47)
High frequency oscillatory ventilation (%)	3 (9)
**Disease severity**	
Asymptomatic (%)	5 (16)
Mild (%)	7 (22)
Moderate (%)	4 (12)
Severe (%)	13 (41)
Critical (%)	3 (9)

*GA, gestational age; IQR, interquartile range; s, seconds; INR, international normalized ratio.*

The mode of transmission was classified as possible *in utero* SARS-CoV-2 transmission 6%, possible intrapartum SARS-CoV-2 transmission 3%, and neonatal infection-acquired postpartum 91% (30% community acquired, 60% acquired in the hospital).

The baseline LUS study was performed with a handheld device in 88% of cases (28 patients). LUS with hemodynamic consultation (which includes head ultrasound) were performed in seven cases: (1) 40 weeks with a large patent ductus arteriosus (PDA) and pulmonary hypertension; (2) 39 weeks with diaphragmatic hernia, perinatal asphyxia, and maternal cocaine use; (3) 28 weeks, 32 corrected with ectopic left kidney, renal failure in dialysis, large hemodynamically significant PDA; (4) 40 weeks term infant with respiratory distress, pulmonary hypertension ruled out; (5) 37 weeks, 38 corrected with perinatal asphyxia post-therapeutic hypothermia; (6) 32 weeks with mild to moderate pulmonary hypertension and right germinal matrix/intraventricular hemorrhage with periventricular hemorrhagic infarction; and (7) 37 weeks, 41 corrected treated at birth for neonatal pneumonia with antibiotics for 10 days. A cardiology consult with lung and head ultrasound was performed in 33 weeks with esophageal atresia, partial anomalous pulmonary venous return, and PDA and with severe pneumonia and absent septum pellucidum/corpus callosum hypoplasia (Table 2). The study was performed by a single operator 69%, image acquisition at a distance 28%, and a trained fellow 3%.

**TABLE 2:** Ultrasonographic assessment during the acute phase of the SARS-CoV-2 infection.

Patient/Diagnosis/(*) Deceased	GA	CGA	LUSS	Hemodynamic/Cardiology consultation	Head ultrasound
**First wave**					
1. COVID Pneumonia, Pulmonary hypertension, large PDA, rule out aortic coarctation	40	40	18	Coarctation ruled out. Moderate PDA. Moderate to severe Pulmonary Hypertension	Normal
2. Diaphragmatic hernia, perinatal asphyxia, maternal cocaine use*	39	39	22	Severe pulmonary hypertension, biventricular failure	Large hyperechogenic right parietal-occipital lesion probably ischemic
3. Late preterm, apneas	35	36	6		
4. Gastroschisis	37	42	10		
5. RDS, Pneumomediastinum, IVH	29	33	12		No progression of IVH
6. RDS, Enterocolitis, PDA medical management, severe IVH, moderate BPD	28	45	14		No progression of IVH
7. RDS, Perinatal asphyxia, ectopic left kidney, renal failure on peritoneal dialysis, large PDA*	28	31	22	Moderate to severe pulmonary hypertension, large PDA	Non-hemorrhagic ventricular dilatation
8. RDS, Severe IVH, Ventriculoperitoneal shunt, moderate BPD	25	43	14		
9. Intra uterine growth restriction	30	31	1		
10. RDS, non-hemodynamically significant PDA	29	33	8		
11. Vesical exstrophy, tethered cord	38	43	0		
12. RDS, Perinatal asphyxia, Enterocolitis, Severe IVH, Severe BPD, Severe pulmonary hypertension, right ventricular failure *	26	44	16		
13. Term infant with respiratory distress	39	41	2		
14. RDS, Stickler syndrome, ROP laser therapy	30	39	12		Periventricular leukomalacia grade I
15. RDS, Hospital acquired infection	30	31	14		IVH ruled out
16. Term infant with respiratory distress. COVID Pneumonia	40	40	16	Adequate biventricular function, pulmonary hypertension ruled out	Normal
17. Esophageal atresia, anorectal malformation with fistula, Tetralogy of Fallot	37	38	7	Pulmonary atresia	
18. Late preterm, community acquired COVID infection	35	38	12		
19. Perinatal asphyxia (post therapeutic hypothermia), seizures	37	38	12	Adequate biventricular function, pulmonary hypertension ruled out	Near total asphyxia pattern
**Second wave**					
20. Esophageal atresia, early onset sepsis, COVID pneumonia, acute renal failure, partial anomalous pulmonary venous return, PDA*	33	33	21	Partial anomalous pulmonary venous return, PDA. Moderate to severe pulmonary hypertension	Septum pellucidum agenesis/Corpus callosum hypoplasia
21. RDS, intestinal atresia, early onset sepsis, COVID pneumonia	32	33	20		
22. Anorectal malformation without fistula	37	37	10		
23. RDS, meconial peritonitis, suspected cystic fibrosis	29	29	16		Normal
**Third wave**					
24. Term infant with respiratory distress	38	42	2		
25. Term infant with respiratory distress	37	37	2		
26. RDS, Severe IVH, COVID pneumonia	32	32	16	Mild to moderate pulmonary hypertension	Germinal matrix/intraventricular hemorrhage with periventricular hemorrhagic infarction
27. Hypertrophic pyloric stenosis	37	40	0		
28. Hypernatremic dehydration, acute renal failure	38	39	1		
29. Term infant with respiratory distress	41	44	1		
30. Intestinal malrotation	37	37	1		
31. RDS	31	31	8		Rule out IVH
32. Neonatal pneumonia, COVID pneumonia*	37	41	19	Mild to moderate pulmonary hypertension, normal biventricular function	Normal

*GA, gestational age; CGA, corrected gestational age; LUSS, lung ultrasound semiquantitative score; PDA, patent ductus arteriosus; RDS, respiratory distress syndrome; IVH, intraventricular hemorrhage; BPD, bronchopulmonary dysplasia; ROP, retinopathy of prematurity.*

Patients were studied under mechanical ventilation 47%, indirect oxygen 25%, without oxygen 13%, HFOV 9%, and CPAP 6%. Antibiotics for early-onset sepsis or suspected superimposed bacterial infection were administered in 28%. Hemodynamic support was necessary on 25%.

**FIGURE 2:**
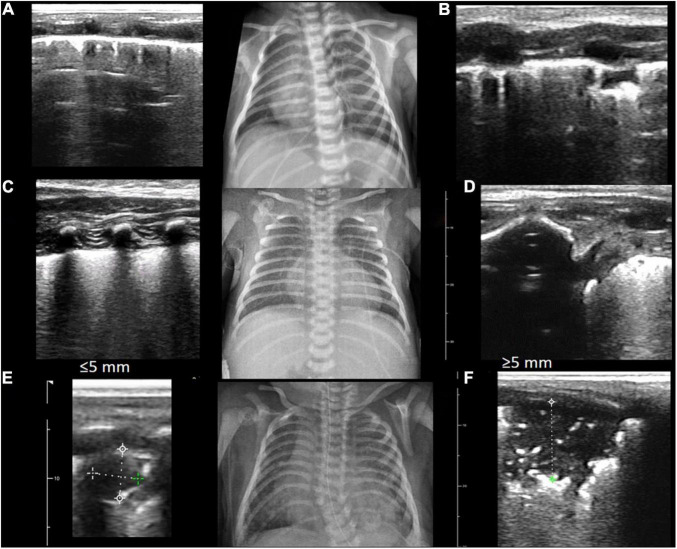
LUS abnormalities and its corresponding radiography are shown. Abnormal patterns found were B-line interstitial pattern 90% **(A)**, irregular/interrupted/thick pleural line 88% **(B)**, compact B-lines 65% **(C,D)**, small consolidations (≤5 mm) 34% **(E)**, and extensive consolidations (≥5 mm) 37% **(F)**.

Lung imaging (radiography or LUS) was abnormal in 75% of babies admitted and 95% of newborns included in this study. LUS abnormalities found were B-line interstitial pattern 90%, irregular/interrupted/thick pleural line 88%, compact B lines 65%, small consolidations (≤5 mm) 34%, and extensive consolidations (≥5 mm) 37%. Consolidations showed posterior predominance (70%) (Figure 2).

All intubated patients were found to have the endotracheal tube *in situ*. After consultation, the recommendation to the attending team was pronation (41%) and increase in positive end expiratory pressure (34%).

**FIGURE 3:**
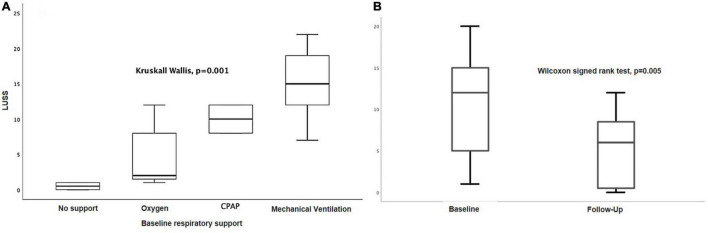
**(A)** LUSS median differences between levels of pulmonary support (Kruskal–Wallis, *p* = 0.001). **(B)** Median difference of baseline and follow-up LUSS (Wilcoxon signed-rank test, *p* = 0.005).

**FIGURE 4:**
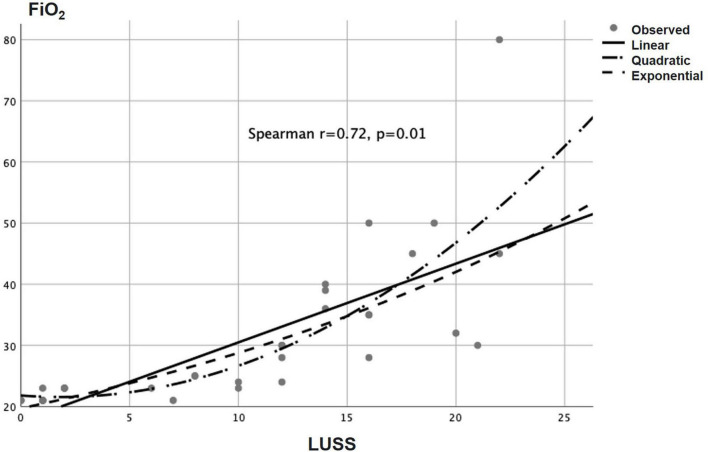
Correlation between LUSS and FiO_2_ needed (Spearman *r* = 0.72, *p* = 0.01).

**FIGURE 5:**
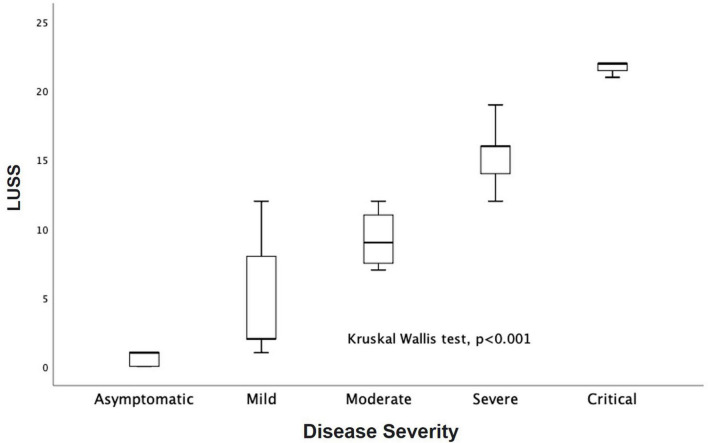
LUSS median differences between disease severity (Kruskal–Wallis, *p* = 0.001).

Baseline LUSS was 11, 2–16. LUSS showed a median difference between levels of pulmonary support (Kruskal–Wallis, *p* = 0.001). For the follow-up LUS, 12 patients were discharged early (two with oxygen, nine without oxygen, and one transfer to another service), and 15 patients were followed. Follow-up LUSS was 6, 0–9. Seven patients were discharged with oxygen and eight without oxygen. A median difference of baseline and follow-up LUSS was demonstrated with the Wilcoxon signed-rank test (*p* = 0.005) (Figure 3). There was a positive correlation between LUSS and FiO_2_ needed (Spearman *r* = 0.72, *p* = 0.01) (Figure 4). A median difference between levels of disease severity (asymptomatic, mild, moderate, severe, and critical) was found (Kruskal–Wallis, *p* = 0.001) (Figure 5).

**FIGURE 6:**
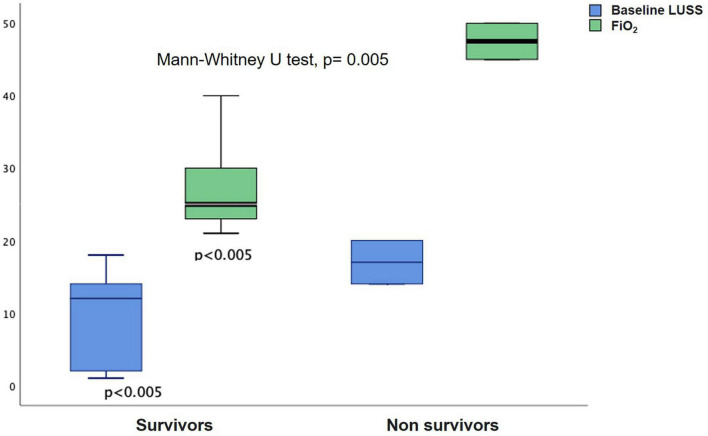
Rank difference between LUSS and FiO_2_ needed between survivors and non-survivors (Mann–Whitney *U*-test, *p* = 0.005).

Five patients died (LUSS 21, 19–22); all of them had comorbidities: (1) 39 weeks with diaphragmatic hernia and perinatal asphyxia; (2) 26 weeks, 44 corrected with severe bronchopulmonary dysplasia with moderate to severe pulmonary hypertension; (3) 28 weeks, 32 corrected with ectopic left kidney, renal failure in dialysis, and large hemodynamically significant PDA; (4) 33 weeks with absent septum pellucidum/corpus callosum hypoplasia, esophageal atresia, and partial anomalous pulmonary venous return; and (5) 37 weeks, 41 corrected treated at birth for neonatal pneumonia with antibiotics for 10 days. Four required the first study to include a hemodynamic/cardiology consult, and one was previously known for pulmonary hypertension (Table 2). A significant rank difference of LUSS and FiO_2_ needed between survivors and non-survivors was found (Mann–Whitney *U*-test, *p* = 0.005) (Figure 6).

Pleural effusion was found in one patient as a surgical complication after an esophageal repair. Thoracocentesis was guided with a handheld device.

## Discussion

During the pandemic, LUS has been shown to help in the detection and staging of diseases. Common patterns described include irregular and thickened pleural line and multiple B-lines ranging from interstitial (three or more well-spaced B-lines) to compact (white lung), demonstrating interstitial to alveolo-interstitial edema. Consolidation is found to be associated with the progression of the disease and its complications (10). LUS has shown a good correlation with computed tomography (11), so it was considered a valid tool during the pandemic. Caroselli et al. found in a systematic review a similar proportion of severe or critical pediatric patients between conventional radiography, CT, and LUS studies, indicating that LUS should be encouraged in pediatric patients, especially in under-resourced areas (12).

Handheld devices have shown been to be accessible, radiation free, and easy to decontaminate (13). As previously described, we found a handheld device to be a useful and affordable option to maintain the point-of-care machine in the NICU, only using it in selected cases.

Neonatal reports are scarce. Feng et al. in China described five cases (1–18 days). Abnormal findings were an abnormal pleural line, B-line interstitial and alveolar syndrome, and a small range of pulmonary consolidations. On follow-up, LUS improved according to patient status (14). Pineda Caplliure et al. in Spain described one case of horizontal transmission in a 10-day neonate. LUS revealed pleural thickening, converging B-lines in the posterior chest, and subpleural consolidation at the lung base level of the right hemithorax (15). Most of our cases were horizontal (community and hospital), and we found the same range of abnormalities (B-line interstitial pattern, irregular/interrupted/thick pleural line, compact B-lines, and consolidations).

A positive correlation between LUSS and FiO_2_ needed was found. LUSS has been shown to correlate with the Silverman Anderson score (16), oxygenation (17), and inflammation in animal (18) and human models (19). Gregorio-Hernández et al. in Spain described one preterm and two term newborns with baseline diagnosis of meconium aspiration syndrome, bronchopulmonary dysplasia, and Hirschsprung’s disease. LUS showed B-lines, consolidation, and spared areas. Using LUSS, they describe the progression of respiratory involvement (20). In our study, we found differences between LUSS and respiratory support needed on the basal study as well as an improvement in follow-up, considering that newborns included had baseline diagnosis and risk factors to have an abnormal LUS. Li et al. in Wuhan, China, presented a case age- and gender-matched control study (11 newborns per arm). LUSS was acquired by assessing a 3-point scale in 12 lung regions per subject. Compared with controls, COVID-19 neonates showed sparse or confluent B-lines, disappearing A-lines, abnormal pleural line, and consolidations. The LUSS was significantly higher in the COVID-19 group. Most of the lesions were in the bilateral inferior and posterior regions (21). In our study, we also found a posterior predominance of consolidations (70%).

Matsuoka et al., in Brazil, published a pictorial essay of 27 neonates (26–38 weeks), comparing COVID-19-negative patients with respiratory symptoms and COVID-19 patients with and without respiratory symptoms (number of patients per group non-specified). They found similar images between groups, concluding that LUS was efficient in evaluating COVID-19-related changes, as well as pathologies inherent to the neonatal period (22).

On three cases, early symptomatology was shown (*in utero* SARS-CoV-2 transmission 6% and possible intrapartum SARS-CoV-2 transmission 3%). Although vertical transmission has been documented, it is not common. Severe placental damage induced by the virus may be detrimental for the neonate independently of vertical transmission; the three patients mentioned showed pulmonary hypertension and high LUSS; one patient died (23).

Pleural effusion has been described in pediatric COVID case series (24); we only found it as a surgical complication in a follow-up study of a baby that developed chylothorax after an esophageal repair surgery. A handheld device was used to guide the thoracocentesis.

Five patients died, all with previous severe health conditions. Deceased patients showed higher LUSS and FiO_2_ needs, and all required a hemodynamic/cardiology consult, reflecting its severity at baseline. It has been difficult in the literature to find “pure” severe neonatal COVID cases; most patients have comorbidities and respiratory symptoms, representing a mixture of baseline diagnosis and superimposed COVID infection.

It has been shown that in viral infections, LUS findings are non-specific. Descriptions include non-homogeneous lungs with pleural line abnormalities (pleural line thickening and/or irregularities, smaller “subpleural” consolidations), areas with multiple B-lines, and larger consolidations (25). In the case of newborns, the effect of the viral infection is frequently superimposed with the parenchymal baseline disease depending on gestational age and clinical history.

As shown by Musolino et al. in children, LUS findings are associated with COVID-19 disease severity (26). In our study, we used the Parri et al. scale (9), taking into account some adaptations as many of our patients had baseline diagnosis and acquired the infection in the community or had a healthcare-associated infection. An association between LUSS and disease severity was found as well as a decrease with patient improvement. This highlights one of the main advantages of LUS, that is, being a useful, feasible, repeatable, and safe tool for the clinician, complementing clinical evaluation and with the advantage of allowing follow-up without ionizing radiation.

Diaphragmatic ultrasound has been shown to be useful in classifying the severity of viral infections; specifically, in the case of bronchiolitis, pediatric patients in an emergency department with lower values of diaphragmatic thickening fraction required high-flow nasal cannula (27). Adding diaphragmatic excursion as well as thickness might be useful; nevertheless, as our baseline studies were performed with a handheld linear device, it was not included in our studies.

Our study has several limitations as it was designed and performed during the pandemic where allocation and discharge policies changed over time. Studies were performed as soon as the patient was positive with an RT-PCR performed in our institution. Timing of clinical assessment and imaging studies depended on adequate personal protective equipment availability and were performed according to patient needs. Secondary to hospital policies, only one RT-PCR was performed, and the patient was classified as COVID-19 completing isolation time in order to return to the general guard (open NICU). As we represent one of the main reference third-level care in Mexico City and receive patients from all over the republic, baseline diagnosis was diverse and the population was heterogenous, ranging from preterm infants to surgical patients with congenital anomalies. This did not allow specific design and specific patient-type stratification, so all consecutive positive patients were included. Posterior field score might be overestimated as the whole study was performed in one time because, during the pandemic, patient handling and protective equipment was limited. Routinely, when we examine the anterior and posterior fields as when looking for consolidations, we normally examine the anterior field with the baby lying prone, and then 1 h later, we investigate the posterior field according to the findings reported by Louis et al. (28).

The strength of the study is that it represents a real-world depiction of neonatal patients during the acute phase of the pandemic in a middle-income country referral center. Baseline diagnosis can cause several of the LUS findings, but the correlation of LUSS with FiO_2_ needed, the differences between disease severity and ventilatory support groups, and the decrease with patient improvement show its usefulness as a monitoring tool. As shown by different publications, LUS is a useful tool in clinical examination and can quantify the loss of aeration at the bedside (29, 30). Sequential assessments can help the clinician identify improvement or worsening; thus, they can be used to classify the severity of the disease and follow its progress (31). Repeatability is a key feature of LUS. Our study was performed by different users, all with basic training but variable experience; nevertheless, all regions were investigated, and diagnosis and scoring were feasible in our unit (3 years of experience prior to the pandemic).

## Conclusion

Lung ultrasound patterns found were like the ones described in other series (neonatal and pediatrics) including B-line interstitial pattern, irregular/interrupted/thick pleural line, compact B-lines, and small consolidations and extensive consolidations with posterior predominance. Eighty-eight percent of studies were performed with handheld affordable equipment. While there is no specific pattern, it varies according to gestational age and baseline diagnosis; LUS was shown to be useful in assessing lung involvement. LUSS that correlated with FiO_2_ needs was higher in patients with severe and critical diseases who needed ventilatory support and decreased with patient improvement. Deceased patients had higher LUSS and FiO_2_ needs.

## Data Availability Statement

The original contributions presented in the study are included in the article/supplementary material, further inquiries can be directed to the corresponding author/s.

## Ethics Statement

The studies involving human participants were reviewed and approved by the Ethics/Research Committee: HIM-2020-048. Written informed consent to participate in this study was provided by the participants’ legal guardian/next of kin. Written informed consent was obtained from the individual(s), and minor(s)’ legal guardian/next of kin, for the publication of any potentially identifiable images or data included in this article.

## Author Contributions

DI-R designed the study, performed ultrasounds, and collected the clinical data. AE-E, ES-M, and AM-V performed ultrasounds and collected the clinical data. DV-G and EV-S collected the clinical data and revised the manuscript. HM-G designed the study and performed the statistical analysis. All authors read and approved the final version of the manuscript.

## Conflict of Interest

The authors declare that the research was conducted in the absence of any commercial or financial relationships that could be construed as a potential conflict of interest.

## Publisher’s Note

All claims expressed in this article are solely those of the authors and do not necessarily represent those of their affiliated organizations, or those of the publisher, the editors and the reviewers. Any product that may be evaluated in this article, or claim that may be made by its manufacturer, is not guaranteed or endorsed by the publisher.

## References

[1] González-García N Castilla-Peón MF Solórzano Santos F Jiménez-Juárez RN Martínez Bustamante ME Minero Hibert MA Covid-19 incidence and mortality by age strata and comorbidities in Mexico city: a focus in the pediatric population. *Front Public Health.* (2021) 9:738423. 10.3389/FPUBH.2021.738423 34568267 PMC8459904

[2] Raschetti R Vivanti AJ Vauloup-Fellous C Loi B Benachi A de Luca D. Synthesis and systematic review of reported neonatal SARS-CoV-2 infections. *Nat Commun.* (2020) 11:5164. 10.1038/S41467-020-18982-9 33060565 PMC7566441

[3] Wang M Luo X Wang L Estill J Lv M Zhu Y A comparison of lung ultrasound and computed tomography in the diagnosis of patients with COVID-19: a systematic review and meta-analysis. *Diagnostics (Basel, Switzerland).* (2021) 11:1351. 10.3390/DIAGNOSTICS11081351 34441286 PMC8394642

[4] Giorno EPC de Paulis M Sameshima YT Weerdenburg K Savoia P Nanbu DY Point-of-care lung ultrasound imaging in pediatric COVID-19. *Ultrasound J.* (2020) 12:50. 10.1186/S13089-020-00198-Z 33252715 PMC7702205

[5] Raimondi F Yousef N Migliaro F Capasso L de Luca D. Point-of-care lung ultrasound in neonatology: classification into descriptive and functional applications. *Pediatric Res.* (2021) 90:524–31. 10.1038/S41390-018-0114-9 30127522 PMC7094915

[6] Villa-Guillén M Garduño-Espinosa J Herrera-Segura MG Moreno-Espinoza S de la Rosa-Zamboni D López-Martínez B Restructuring of a pediatric hospital in the face of the COVID-19 pandemic. *Boletin Medico Del Hospital Infantil De Mexico.* (2020) 78:3–9. 10.24875/BMHIM.20000265 33226975

[7] De Rose C Inchingolo R Smargiassi A Zampino G Valentini P Buonsenso D. How to Perform pediatric lung ultrasound examinations in the time of COVID-19. *J Ultrasound Med Off J Am Institute Ultrasound Med.* (2020) 39:2081–2. 10.1002/JUM.15306 32320081 PMC7264804

[8] Kim DJ Jelic T Woo MY Heslop C Olszynski P. Just the Facts: recommendations on point-of-care ultrasound use and machine infection control during the coronavirus disease 2019 pandemic. *CJEM.* (2020) 22:445–9. 10.1017/CEM.2020.364 32268930 PMC7188692

[9] Parri N Magistà AM Marchetti F. Characteristic of COVID-19 infection in pediatric patients: early findings from two Italian Pediatric Research Networks. *Eur J Pediatrics.* (2020) 179:1315–23. 10.1007/s00431-020-03683-8 32495147 PMC7269687

[10] Musa MJ Yousef M Adam M Wagealla A Boshara L Belal D The role of lung ultrasound before and during the COVID-19 pandemic: a review article. *Curr Med Imag.* (2021) 17: [Online ahead of print], 10.2174/1573405617666211006122842 34620067

[11] Chiumello D Mongodi S Algieri I LucaVergani G Orlando A Via G Assessment of lung aeration and recruitment by CT scan and ultrasound in acute respiratory distress syndrome patients. *Crit Care Med.* (2018) 46:1761–8. 10.1097/CCM.0000000000003340 30048331

[12] Caroselli C Blaivas M Falzetti S. Diagnostic imaging in newborns, children and adolescents infected with severe acute respiratory syndrome coronavirus 2 (SARS-CoV-2): is there a realistic alternative to lung High-Resolution Computed Tomography (HRCT) and chest X-Rays? A systematic review of the literature. *Ultrasound Med Biol.* (2021) 47:3034–40. 10.1016/J.ULTRASMEDBIO.2021.07.015 34429231 PMC8302856

[13] Haji-Hassan M Lenghel LM Bolboacã SD. Hand-Held ultrasound of the lung: a systematic review. *Diagnostics (Basel, Switzerland).* (2021) 11:1381. 10.3390/DIAGNOSTICS11081381 34441315 PMC8392700

[14] Feng XY Tao XW Zeng LK Wang WQ Li G. [Application of pulmonary ultrasound in the diagnosis of COVID-19 pneumonia in neonates]. *Zhonghua Er Ke Za Zhi = Chin J Pediatrics.* (2020) 58:347–50. 10.3760/CMA.J.CN112140-20200228-00154 32392948

[15] Pineda Caplliure A Porcar Almela M Navarro Albert A Muñoz Vicente E Mansilla Roig B. Usefulness of chest ultrasound in a neonatal infection due to SARS-CoV-2. *Anal Pediatria.* (2021) 94:412–3. 10.1016/J.ANPEDE.2021.04.001 33903852 PMC8059871

[16] Raimondi F Yousef N Rodriguez Fanjul J de Luca D Corsini I Shankar-Aguilera S A multicenter lung ultrasound study on transient tachypnea of the neonate. *Neonatology.* (2019) 115:263–8. 10.1159/000495911 30731475

[17] Brat R Yousef N Klifa R Reynaud S Shankar Aguilera S de Luca D. Lung ultrasonography score to evaluate oxygenation and surfactant need in neonates treated with continuous positive airway pressure. *JAMA Pediatrics.* (2015) 169:e151797. 10.1001/JAMAPEDIATRICS.2015.1797 26237465

[18] Elsayed YN Hinton M Graham R Dakshinamurti S. Lung ultrasound predicts histological lung injury in a neonatal model of acute respiratory distress syndrome. *Pediatric Pulmonol.* (2020) 55:2913–23. 10.1002/PPUL.24993 32741109 PMC7436735

[19] Yousef N Vigo G Shankar-Aguilera S de Luca D. Semiquantitative ultrasound assessment of lung aeration correlates with lung tissue inflammation. *Ultrasound Med Biol.* (2020) 46:1258–62. 10.1016/J.ULTRASMEDBIO.2020.01.018 32081586

[20] Gregorio-Hernández R Escobar-Izquierdo AB Cobas-Pazos J Martínez-Gimeno A. Point-of-care lung ultrasound in three neonates with COVID-19. *Eur J Pediatrics.* (2020) 179:1279–85. 10.1007/S00431-020-03706-4 32504135 PMC7274567

[21] Li W Fu M Qian C Liu X Zeng L Peng X Quantitative assessment of COVID-19 pneumonia in neonates using lung ultrasound score. *Pediatric Pulmonol.* (2021) 56:1419–26. 10.1002/PPUL.25325 33713586 PMC8250904

[22] Matsuoka MW da Rocha SMS Gibelli MABC Nicolau CM de Carvalho WB Suzuki L. Use of lung ultrasound in neonates during the COVID-19 pandemic. *Radiol Brasileira.* (2020) 53:401–4. 10.1590/0100-3984.2020.0110 33304008 PMC7720664

[23] Cribiù FM Erra R Pugni L Rubio-Perez C Alonso L Simonetti S Severe SARS-CoV-2 placenta infection can impact neonatal outcome in the absence of vertical transmission. *J Clin Investigat.* (2021) 131:e145427. 10.1172/JCI145427 33497369 PMC7954587

[24] Guitart C Suárez R Girona M Bobillo-Perez S Hernández L Balaguer M Lung ultrasound findings in pediatric patients with COVID-19. *Eur J Pediatrics.* (2021) 180:1117–23. 10.1007/S00431-020-03839-6 33089388 PMC7577846

[25] Yousef N De Luca D. The role of lung ultrasound in viral lower respiratory tract infections. *Am J Perinatol.* (2018) 35:527–9. 10.1055/s-0038-1637758 29694988

[26] Musolino AM Supino MC Buonsenso D Papa RE Chiurchiù S Magistrelli A Lung ultrasound in the diagnosis and monitoring of 30 children with coronavirus disease 2019. *Pediatric Pulmonol.* (2021) 56:1045–52. 10.1002/ppul.25255 33404197

[27] Buonsenso D Supino MC Giglioni E Battaglia M Mesturino A Scateni S Point of care diaphragm ultrasound in infants with bronchiolitis: a prospective study. *Pediatric Pulmonol.* (2018) 53:778–86. 10.1002/ppul.23993 29578644

[28] Louis D Belen K Farooqui M Idiong N Amer R Hussain A Prone versus supine position for lung ultrasound in neonates with respiratory distress. *Am J Perinatol.* (2021) 38:176–81. 10.1055/s-0039-1695776 31480084

[29] Ravetti CG Vassallo PF de Barros GM Rocha GC Chamon S Borges IN Lung ultrasound can predict the clinical course and severity of COVID-19 disease. *Ultrasound Med Biol.* (2021) 47:2090–6. 10.1016/j.ultrasmedbio.2021.04.026 34088531 PMC8092622

[30] Chardoli M Sabbaghan Kermani S Abdollahzade Manqoutaei S Loesche MA Duggan NM Schulwolf S Lung ultrasound in predicting COVID-19 clinical outcomes: a prospective observational study. *J Am College Emerg Phys Open.* (2021) 2:e12575. 10.1002/emp2.12575 34755148 PMC8560933

[31] Buonsenso D Parri N De Rose C Valentini P Gemelli-pediatric Covid-19 team. Toward a clinically based classification of disease severity for paediatric COVID-19. *Lancet Infect Dis.* (2021) 21:22. 10.1016/S1473-3099(20)30396-0 PMC7228718 32422205

